# Gastric Emptying and Gastrointestinal Transit Compared among Native and Hydrolyzed Whey and Casein Milk Proteins in an Aged Rat Model

**DOI:** 10.3390/nu9121351

**Published:** 2017-12-13

**Authors:** Julie E. Dalziel, Wayne Young, Catherine M. McKenzie, Neill W. Haggarty, Nicole C. Roy

**Affiliations:** 1 Food Nutrition & Health Team, Food & Bio-Based Products Group, AgResearch, Grasslands Research Centre, Palmerston North 4442, New Zealand; wayne.young@agresearch.co.nz (W.Y.); nicole.roy@agresearch.co.nz (N.C.R.); 2 Riddet Institute, Massey University, Palmerston North 4442, New Zealand; 3 High Value Nutrition, National Science Challenge, Liggins Institute, The University of Auckland, Auckland 1142, New Zealand; 4 Bioinformatics and Statistics, AgResearch, Grasslands Research Centre, Palmerston North 4442, New Zealand; catherine.mckenzie@agresearch.co.nz; 5 Fonterra Co-Operative Group, Palmerston North 4442, New Zealand; neill.haggarty@fonterra.com

**Keywords:** colon, fecal output, motility, opioid, serotonin, elderly

## Abstract

Little is known about how milk proteins affect gastrointestinal (GI) transit, particularly for the elderly, in whom digestion has been observed to be slowed. We tested the hypothesis that GI transit is faster for whey than for casein and that this effect is accentuated with hydrolysates, similar to soy. Adult male rats (18 months old) were fed native whey or casein, hydrolyzed whey (WPH) or casein (CPH), hydrolyzed blend (HB; 60% whey:40% casein), or hydrolyzed soy for 14 days then treated with loperamide, prucalopride, or vehicle-control for 7 days. X-ray imaging tracked bead-transit for: gastric emptying (GE; 4 h), small intestine (SI) transit (9 h), and large intestine (LI) transit (12 h). GE for whey was 33 ± 12% faster than that for either casein or CPH. SI transit was decreased by 37 ± 9% for casein and 24 ± 6% for whey compared with hydrolyzed soy, and persisted for casein at 12 h. Although CPH and WPH did not alter transit compared with their respective intact counterparts, fecal output was increased by WPH. Slowed transit by casein was reversed by prucalopride (9-h), but not loperamide. However, rapid GE and slower SI transit for the HB compared with intact forms were inhibited by loperamide. The expected slower GI transit for casein relative to soy provided a comparative benchmark, and opioid receptor involvement was corroborated. Our findings provide new evidence that whey slowed SI transit compared with soy, independent of GE. Increased GI transit from stomach to colon for the HB compared with casein suggests that including hydrolyzed milk proteins in foods may benefit those with slowed intestinal transit.

## 1. Introduction

Milk is a widely consumed natural beverage renowned as a nutritious protein source [1]. Research has revealed additional health properties that may be conferred via the pharmacological actions of digested milk peptides on cardiovascular function, regulation of food intake, and metabolism [2,3,4]. Milk proteins make up 3.5% of cows’ milk, consisting of 80% caseins and 20% whey proteins [5]. Casein and whey are used in different food products for specific uses; for example, casein is the major component of cheese. Whey protein in the concentrated form is used as a dietary supplement for those needing an easily digestible nutritious protein source including, the elderly, infants, and athletes.

During gastrointestinal (GI) digestion, the behaviour of milk proteins differs, with casein considered as a slowly digestible protein and whey a rapidly digestible protein [6]. Whey is rapidly expelled from the stomach, whereas caseins precipitate in the low pH of the stomach and coagulate, slowing gastric emptying (GE). Size, structure, and solubility of casein micelles in the stomach affect digestibility and alter GI transit. Although casein has long been known to slow both GE (due to curd formation) and small intestinal (SI) transit, these effects may be altered by specific hydrolysis methods. Casein is a rich source of peptides released upon hydrolysis or during GI digestion that include angiotensin-converting enzyme inhibitors, opioids, and antimicrobial peptides [2]. Some of these peptides have been shown to affect GI transit (due to the presence of opioid peptides) [7,8,9]. Modulation of GI transit rate by peptides has the potential for functional benefits through slower small intestine transit, allowing greater absorption time for nutrients, or increased transit to reduce constipation.

The effect of the hydrolysis of dairy proteins on GI motility to influence the transit of contents is not well understood. By determining how hydrolysis of different types of dairy protein affects GE and GI transit will provide better information on health effects of potential functional foods. A number of GI disorders that affect gastric and colonic motility occur more frequently in the aging population [10], including slowed gastric emptying [11] and constipation [12,13]. Slowed gastric emptying with aging can affect regulation of appetite, postprandial glycemia, and blood pressure [11]. More generally, delayed GE can result in early satiety, bloating, pain or discomfort, malnutrition, and weight loss [14,15]. Functional dairy foods may assist self-management of mild dysmotility, as occurs in functional gastrointestinal disorders, to increase or decrease transit of intestinal contents. For example, in the elderly, slower small intestine transit might assist nutrient uptake, whereas faster colonic transit may help reduce constipation.

While whey reaches the jejunum faster than casein, hydrolysis is slower, which is thought to allow for greater absorption over the length of the SI [16]. Whether this is due to increased digestibility of whey or slower for casein GE is unknown [17]. The ability of whey to alter transit throughout the GI tract is also less understood than for casein. Whey protein hydrolysate (WPH) protein has a nutritionally available amino acid composition. Both WPH and intact whey are used as protein supplements for the elderly. This is because whey affects a range of biological processes associated with aging in humans: antimicrobial activity, immune modulation, improved muscle strength and body composition [18], as well as protection against cardiovascular disease, osteoporosis, cancer, hypertension. It also has cholesterol-lowering and mood-enhancing properties [2,16,19,20,21]. Despite the widespread use of whey and casein milk proteins, studies directly comparing the effect of their hydrolysis on GI transit and their modes of action via receptor pathways with aging are lacking.

Because composition and processing of milk proteins has an impact on their digestion and absorption [22,23,24], combining specific milk protein components provides a way to potentially maximize benefits for digestive health. In addition, milk proteins are digested during the various stages of human GI tract digestion to give rise to an array of peptides that can elicit a variety of physiologic effects that affect the rate of digestion and transit for casein and whey proteins [2,22,25,26]. Some of these peptides modulate opioid [26,27,28,29] or serotonin receptors [30]. Whey supplementation may confer benefits for modulation of GI motility in the elderly. As an example, slowed GI transit resulting in constipation affects 25–30% of people over 65 years of age [12,13], and foods which target this will be of considerable benefit. Because large intestine (LI) transit is slower in aged than in young [31,32] rats, they are used as a model for mature to elderly humans. Gastric emptying also slows with age in rats [33,34], which matches the changes that occur in elderly humans [10,35]. 

The aim of this study was to investigate how whey and casein milk proteins affect GE and GI transit in an aged rat model [36] and whether this differs when the proteins are pre-hydrolyzed. We hypothesized that GI transit is faster in response to whey than for casein, and that this effect is enhanced with hydrolyzates. We therefore compared the effect of casein, whey, CPH, and WPH, on the rate of GI transit of solids in an aged rat model, using hydrolyzed soy as an example of rapid transit and intact casein an example of slow transit. We also wanted to compare casein and whey with their hydrolyzed counterparts, CPH and WPH, as protein sources to discriminate GE and GI transit differences. We also compared a combination formula composed of partially hydrolyzed 60:40 whey/casein, renowned for its nutritional properties and ease of digestion, to determine its potential as a supplement for the elderly by investigating its actions on GI transit [37]. We also wanted to determine whether casein would slow GE and GI transit in this model as expected and therefore provide a benchmark for measuring other milk proteins in this aged animal model. 

The ability of specific modulators to reduce the transit changes induced by the milk proteins was investigated using loperamide (a mu opioid agonist which inhibits GI transit) and prucalopride (a serotonin type 4 receptor agonist which increases GE and colonic transit) [36]. This was to show whether any milk-protein-induced changes detected for GI transit were reversible and to suggest the neural pathways involved.

Rats were the animal model of choice because many dietary preclinical studies are done in this species and pharmacological modulatory doses have been determined [36]. To track the transit of solid contents along the GI tract, we measured the movement of six metallic beads over 12 h from the stomach to the large intestine by high resolution X-ray imaging [36,38]. This technique utilises a barium slurry providing a mix of solid and semi-solid gastric contents, similar to human measurement techniques [15]. This method using metallic beads as markers for transit of solid contents has been validated in two previous studies in which the beads were found in the fecal pellets [36,38].

## 2. Materials and Methods

### 2.1. Animals

This study was conducted following ethical approval (AE12933) by the AgResearch Grasslands Animal Ethics Committee (Palmerston North, New Zealand (NZ)) in accordance with the Animal Welfare Act, 1999 (NZ). Male Sprague Dawley rats were bred at the AgResearch Ruakura Small Animal Unit (Hamilton, New Zealand) and raised in group housing with littermates to 18 months of age (804 ± 13 g). The animals were housed at a constant temperature of 21 °C and maintained under a light/dark cycle (06:00/18:00) in sawdust-lined plastic or stainless steel cages, with food and water provided ad libitum. They were fed a normal rat chow pellet soy-based diet (Prolab^®^ RMH 1800, LabDiet, St. Louis, MO, USA) until seven days prior to the study when they were individually caged and switched to a hydrolyzed soy-based diet (OpenStandard Rodent Diet, Research Diets, Inc., New Brunswick, NJ, USA) in powdered form, for compatibility with metabolic cage requirements. The animals were monitored three times weekly for weight, food intake, and General Health Score (1–5; NZ Animal Health Care Standard). Cages and feeding and drinking containers were cleaned and sterilized weekly. Twenty-eight out of 226 rats were excluded from the study and euthanized due to age-related health issues including: weight loss, lethargy, excessive drinking, swollen or inflamed tissue not cured with antibiotic treatment, or invasive tumours. At the end of the study, all remaining rats were euthanized using carbon dioxide inhalation overdose.

### 2.2. Study Design

**Figure 1:**
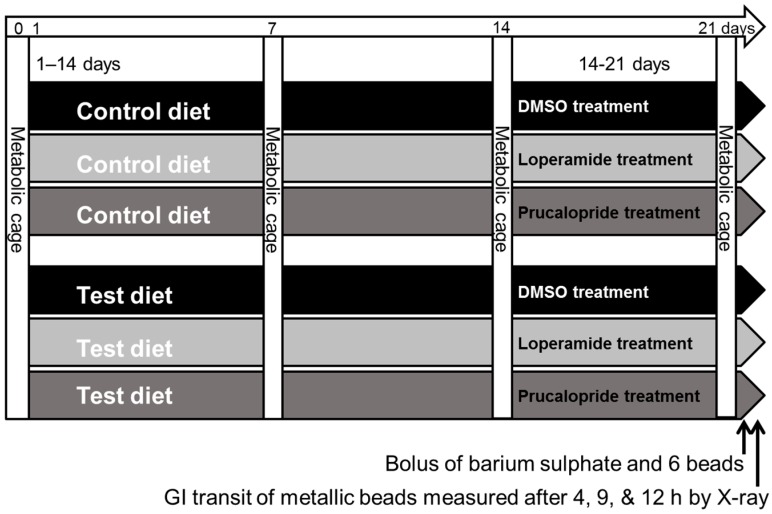
Study design shown for the dietary control (hydrolyzed soy) and one of the five milk protein test diets. GI, gastrointestinal; DMSO, dimethyl sulfoxide.

This study was designed to include one control group and five test diet treatment groups that were also treated with modulatory drugs, either 4 mg/kg/day prucalopride or 1 mg/kg/day loperamide or dimethyl sulfoxide (DMSO) control carrier (Figure 1). The study was carried out as a block design as six blocks of ~36 animals, with age and weight balanced among treatment groups (i.e., 18 groups × 12 animals per group), which resulted in 9–13 animals for each diet plus the drug treatment group.

The drug doses were previously determined to be effective over seven days [36]. Rats were fed the powdered treatment diet for three weeks (day 0 to day 22) and treated with modulatory drugs over the third week (days 15–22). Four days prior to the start of the study, the rats were acclimatized to metabolic cages for 1 h to reduce possible stress symptoms. Rats were individually placed in metabolic cages for 24 h (from 10 am to 10 am) on days 0, 7, 14, and 21, after which food intake and fecal and urine output, were measured.

### 2.3. Dietary Protein

Milk proteins were provided by Fonterra Co-operative Group. Animals were treated with diets in which protein was 20% of the diet (energy) provided as hydrolyzed soy for the control treatment group, and the following milk protein products for the five test diet treatment groups: casein (sodium caseinate 1800), whey (lactic whey protein concentrate WPC7009), partially hydrolyzed CPH (MPH948, degree of hydrolysis (DH) 7.6%), partially hydrolyzed WPH (WPH917, DH 5.0%), partially hydrolyzed HB of 60:40 whey/casein (MPH942, DH 12–17%). The protein was added to a nutritionally balanced protein-free base diet (OpenStandard Modified Rodent Diet, Research Diets, Inc. New Brunswick, NJ, USA) of final composition (kcal %): 15% fat (soybean oil), 65% carbohydrate (cornstarch, maltodextrin 10, dextrose), cellulose, BW200, inulin, minerals, and vitamins.

### 2.4. Pharmacological Treatments

Loperamide hydrochloride (S2480) and prucalopride (S2875) were from Selleck Chemicals (Houston, TX, USA). Animals were administered 4 mg/kg/day prucalopride or 1 mg/kg/day loperamide (in 100% DMSO) or DMSO vehicle only (control) for seven days via a subcutaneous 2 mL capacity slow release osmotic mini-pump (Durect Corporation, Alzet Osmotic Pumps, Cupertino, CA, USA), while controls received DMSO vehicle only via the same delivery method. The surgical implantation procedure has been described previously [36]. Continuous dosing overcame rapid metabolism of prucalopride in rats [39] and avoided frequent subcutaneous injections for loperamide (half-life 9–14 h). Each compound was dissolved in DMSO to give stocks of 20 mg/mL that were diluted in DMSO to give the correct concentration in the mini-pump to deliver the appropriate dose per weight of rat at a flow rate of 10 µL/h.

### 2.5. GI transit Procedures and Measurements

Non-fasted rats were used to maintain normal digestion and transit, and to avoid retention of beads in the stomach as has been reported for solid capsules in fasted rats [40]. The methods used for measuring GI transit have been described previously [36,38]. Briefly, each rat received six solid stainless steel beads, d = 1.4 mm (Bal-tec, Los Angeles, CA, USA), via oral gavage in 2 mL of 15% barium sulfate (E-Z-HD 98% *w/w*, Cat. No. 764, E-Z-EM Canada Inc., provided by Palmerston North Hospital, Palmerston North, New Zealand). Isoflurane anesthesia was induced in a chamber and persisted for 5 min, during which gavage was performed upon recovery of the swallow reflex.

#### 2.5.1. X-ray Imaging

GI transit was tracked at three time points by X-ray imaging under brief isoflurane anesthesia to monitor: exit from stomach (4 h), SI transit (9 h), and LI transit (12 h). The 12 h time point was carried out post-mortem. The metallic beads were visualized by X-ray and the relatively opaque barium sulfate outlined the GI, enabling identification of bead location. Ventral and right lateral views were taken using a portable X-ray unit (Porta 100 HF 2.0 kW High Frequency, Job Corporation, Yokohama, Japan) including: camera and digital cassette (Canon 55G DR sensor panel, Melville, NY, USA) in conjunction with a laptop computer (Lenovo ThinkPad W530, Morrisville, NC, USA). Image files (DICOM) were visualized using MicroDicom DICOM Viewer v8.7 (Simeon Antonov Stoykov, Sofia, Bulgaria). 

#### 2.5.2. Gastric Emptying

Comparative measures of GE were obtained by determining the proportion of animals in which all six beads had exited the stomach for a given treatment over time.

#### 2.5.3. GI transit Score

The rating scale used to classify GI bead location comprised six beads, each given a numeric score depending on its location within the GI tract: (0) stomach; (1) proximal SI; (2) distal SI; (3) cecum; (4) colon; or (5) expelled. The total transit score was the sum of the individual bead scores (maximum = 30 if all expelled). The experimenter was blinded to treatment. A transit score of 3–4 means that on average half of the beads were in the proximal SI. Scores of 10–14 place most of the beads in the distal SI, whereas 16–20 places most in the cecum and 20–22 places half of them as distal as the colon [36,38].

#### 2.5.4. Colonic Transit

The number of beads per rat that had moved from the cecum to the colon or rectum over 3 h was compared. The movement of beads between 9 h (when the majority were in the cecum or distal SI) and 12 h (when a proportion had moved to the colon or rectum) was measured to assess possible differences between treatments in the probability that a bead had transited into the colon by 12 h.

#### 2.5.5. Bead Transit through Colon

The average bead distance travelled along the colon for the beads that had transited from the cecum to the colon over 9–12 h was calculated as a percent of the LI length from the cecum or colon junction to the anus.

### 2.6. Statistical Analysis

All analyses were carried out using GenStat version 17 (VSN International Limited, Hemel Hempstead, UK) or Minitab 17 Statistical software (Minitab Inc., State College, PA, USA). Results are expressed as the mean ± standard error of the mean (SEM).

#### 2.6.1. Animal Metrics

Data were analysed using a Linear Mixed Model via restricted maximum likelihood (REML) for the effect of 14 days of diet treatment only on the difference in the response variable of interest (weight, feed intake, urine output, or fecal output) between day 0 (pretreatment) and day 14 (prior to commencement of pharmacological treatments). To compare among all diet × drug treatments, data were analysed using a Linear Mixed Model (via REML) for the response variable of interest (weight, feed intake, urine output, or fecal output) on day 21, using treatment as factor (18 combinations of drug and diet) and the relevant value on day 14 as covariate. Fisher’s least significant differences were used for the post hoc test.

#### 2.6.2. Transit from Stomach

Logistic regression analyses were carried out at each time point (4, 9, and 12 h) using treatment as the factor, separately to compare differences in the proportion of rats with zero beads in the stomach (0/1).

#### 2.6.3. Transit Score

Changes in GI transit were measured relative to the DMSO/control-treated rats. Due to the expected correlated nature of the data, a linear mixed model (via REML) using drug treatment and diet as factors was used to analyze the data. Fisher’s least significant differences were used for the post hoc test.

#### 2.6.4. Cecum to Colon Transit

Differences in cecum to colon transit were compared using a linear mixed model (via REML) using treatment as factor (18 combinations of drug and diet) and Fisher’s least significant difference post hoc test.

#### 2.6.5. Bead Transit through Colon

Differences in percent distance of bead transit through the colon were compared using a linear mixed model (via REML) using treatment as factor (18 combinations of drug and diet) and Fisher’s least significant difference post hoc test.

## 3. Results

### 3.1. Dietary Effects

Changes in animal metrics are summarized in Table 1.

#### 3.1.1. Body Weight

No physiologically significant differences in body weight (more than 10% difference) occurred from that of 818 ± 14 g on the hydrolyzed soy diet (*n* = 50) between days 0 and 14. However, body weight decreased by 2.2% (18 g) on the casein diet, *p* < 0.01, although only a small change is notable for aged rats where body weight among the other diets varied by less than 1%. 

#### 3.1.2. Food Intake

When on the control hydrolyzed soy diet (day 14), food intake was 26.4 ± 0.7 g/day (*n* = 50). When pre-treatment food intake was compared between days 0 and 14 on the test diets, intake of the casein diet was 11.4% less than the hydrolyzed soy control and less than all the other diets (*p* < 0.05). However, this effect was not detected by day 21.

#### 3.1.3. Fecal Output

Fecal output was not altered between days 0 and 14 on the test diets. By 21 days for control animals receiving DMSO treatment, however, fecal output for WPH was increased by: 50.3% compared with the hydrolyzed soy diet, 42.0% compared with the whey diet, and 74.1% compared with the CPH diet. Fecal output for the HB was increased by 51.0% compared with CPH.

#### 3.1.4. Urine Output

No differences in urine output were detected with any diet × drug treatment between days 0 and 14.

### 3.2. Pharmacological Modulation of Dietary Effects

To determine the effect of the pharmacological treatments on food intake, this was compared among treatments at day 21 (Table 1). 

#### 3.2.1. Food Intake

Loperamide reduced food intake by 15.1% on the hydrolyzed soy diet, by 25.0% on the WPH diet, and by 20.2% on the HB diet, by 18.6% on the CPH diet.

#### 3.2.2. Fecal Output

Loperamide did not alter fecal output on the hydrolyzed soy diet. Loperamide reduced fecal output for WPH by 34.7%. Loperamide reduced fecal output for the whey diet by 35.5% and reduced that for the HB diet by 29.9%. For animals on the CPH diet, prucalopride increased fecal output by 45.4%. It is notable that for animals receiving prucalopride treatment, fecal output was increased for animals on the HB diet by 32.0% compared with those on hydrolyzed soy (also receiving prucalopride).

Twenty-one animals across 14 of the 18 treatment groups (21/198) were excluded from further analysis because no meaningful transit measurements were possible due to GE being substantially delayed over 12 h, as previously reported to occur in 10% of animals using this method [36]. Notably, 13 animals across all loperamide treatment groups were affected but only five were DMSO treated and three were prucalopride-treated animals.

### 3.3. Dietary Effects on Bead Transit (DMSO/Control Treated)

#### 3.3.1. Gastric Emptying

**Figure 2:**
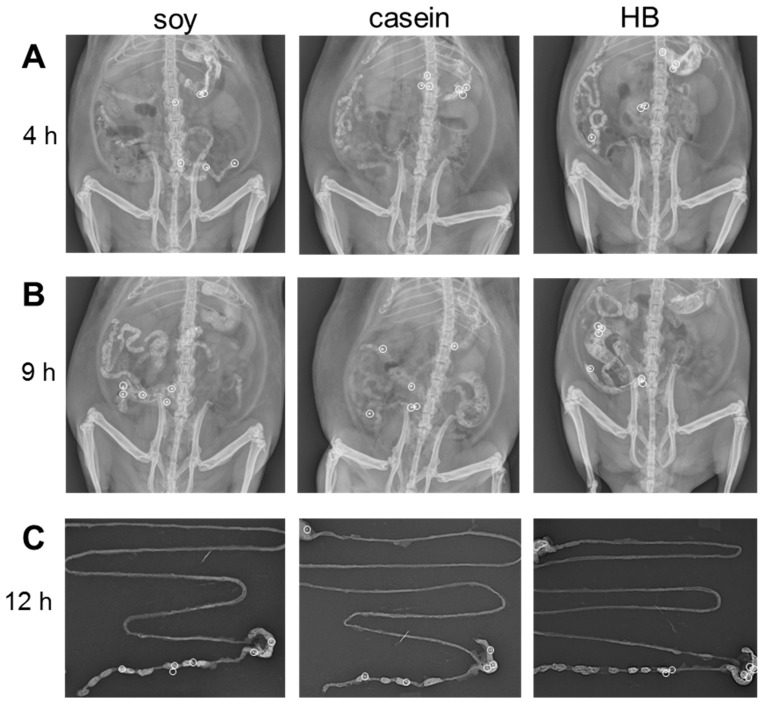
Location of six metallic beads tracked over time for animals treated for soy or milk protein diets. Representative examples of bead location are shown at: (**A**,**B**) 4 h and 9 h in vivo and (**C**) post-mortem GI tract at 12 h of X-ray images in ventral view for animals on soy, casein, and hydrolyzed blend (HB) diets (treated with DMSO).

**Figure 3:**
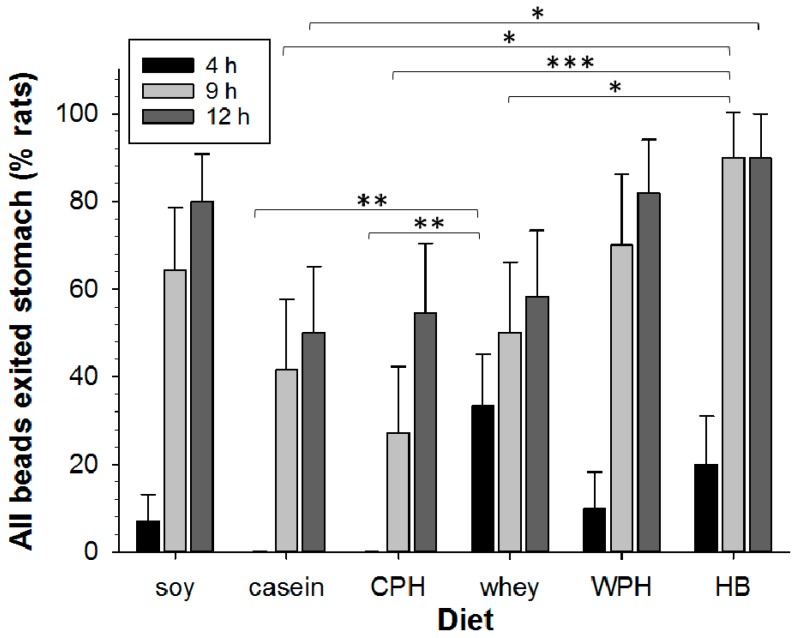
Comparison of gastric emptying (GE) of beads over 12 h for animals treated with soy or milk protein diets (*n* = 8–15 animals per group). The percentage of rats in which all beads had exited the stomach (mean per treatment) is shown at 4 h (black), 9 h (light grey), and 12 h (dark grey) for: soy, casein, hydrolyzed casein (CPH), whey, hydrolyzed whey (WPH), and hydrolyzed blend (HB). Asterisks indicate the significance of each treatment relative to controls (* *p* < 0.05; ** *p* < 0.01; *** *p* < 0.001). Data show mean ± SEM.

For DMSO/control-treated animals on the hydrolyzed soy diet, all beads had exited the stomach in 7% of animals at 4 h post-gavage, 64% at 9 h, and 80% at 12 h (Figure 2 and Figure 3). At 4 h, any delays in GE would be evident. For the casein and CPH, beads remained in the stomach of most animals, indicating a prolonged delay in GE. In contrast, GE at 4 h was 33% more likely to have occurred for whey than for either casein or CPH, yet was similar by 9 h. At 9 h, GE was increased for the HB by 116% compared with casein, 230% compared with CPH, and 80% compared with whey. An 80% difference persisted at 12 h between the HB and casein only. Thus the effect of the proteins on GE may be approximately ranked from rapid to the most delayed: whey > HB > (WPH = hydrolyzed soy) > (casein = CPH).

#### 3.3.2. GI Transit

**Figure 4:**
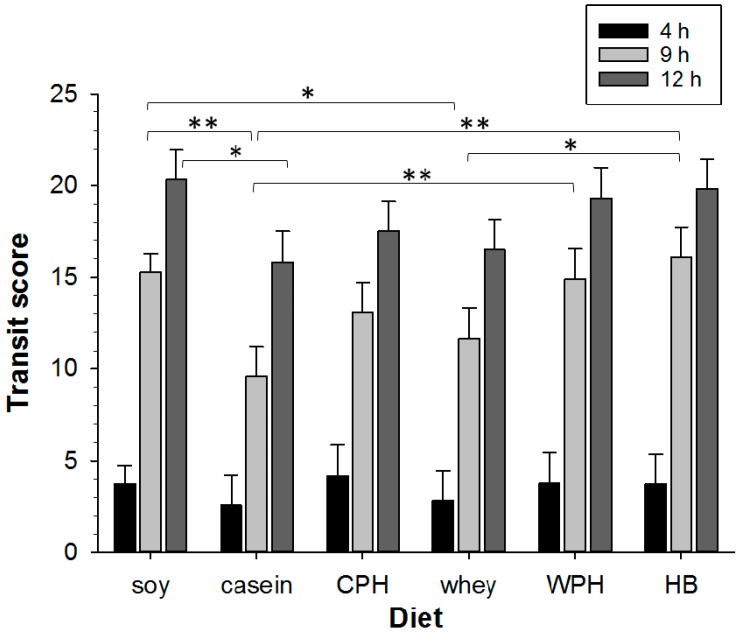
Comparison of GI transit tracked over 12 h for animals treated with soy or milk protein diets (*n* = 1–14 animals per group). Transit scores are shown at 4 h (black), 9 h (light grey), and 12 h (dark grey) for: soy, casein, hydrolyzed casein (CPH), whey, hydrolyzed whey (WPH), and hydrolyzed blend (HB). Asterisks indicate the significance of each treatment relative to controls (* *p* < 0.05; ** *p* < 0.01). Data show mean ± SEM.

At 9 h, the transit score was decreased by 37% for casein and decreased by 24% for whey compared with hydrolyzed soy, and a 22% difference persisted at 12 h for casein but not whey (Figure 4). CPH and WPH did not alter transit compared with their intact protein counterparts, respectively. However, when casein was used as a benchmark, transit at 9 h was increased by 55% for WPH and 68% for the HB. Transit for the HB was increased by 38% compared with whey at 9 h. The effect of the proteins on transit score at 9 h may be approximately grouped and ranked from rapid to slowest transit: (hydrolyzed soy = HB = WPH) ≥ CPH ≥ (whey = casein). Similar to our previous studies, the beads were always found in fecal pellets, providing further validation for this method in monitoring GI transit of solids [36,38].

#### 3.3.3. Caecum to Colon Transit

Bead movement from the cecum at 9 h to the colon at 12 h was compared and no significant differences found. Once in the colon, the percent bead distance travelled along the colon for the beads that had transited from cecum to colon by 12 h did not reveal any diet effect.

#### 3.3.4. Pharmacological Modulation of Bead Transit

**Figure 5:**
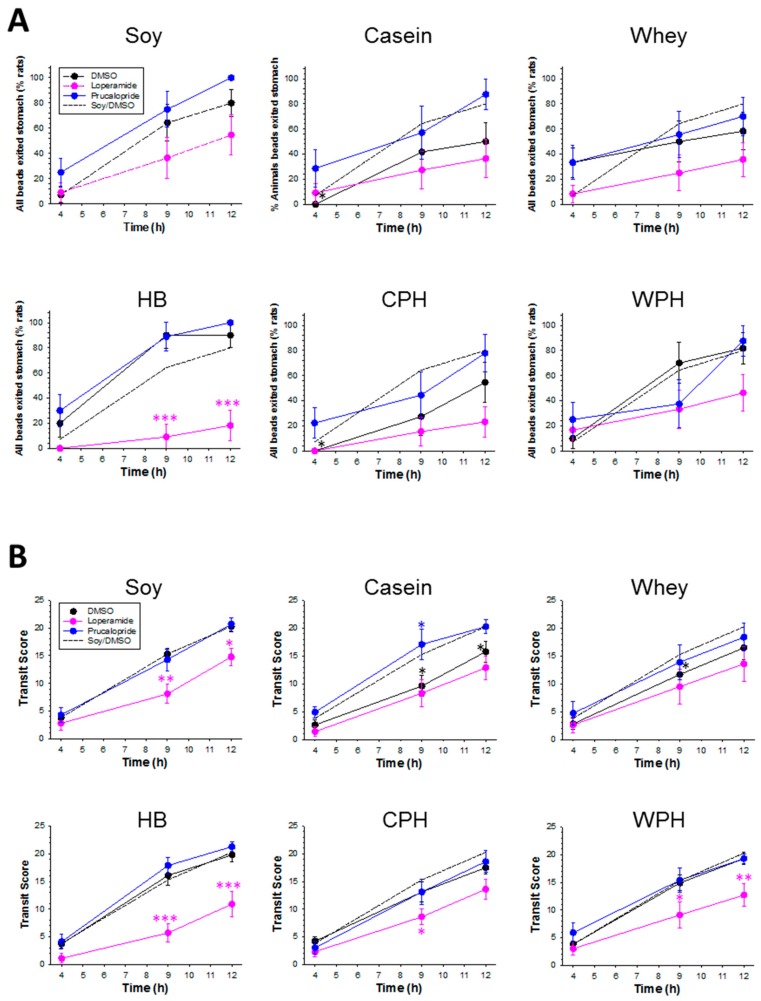
Effect of pharmacological modulators on dietary changes in GE and GI transit for animals treated with soy or milk protein diets. (**A**) The percentage of rats in which all beads had exited the stomach, and (**B**) transit scores for: soy, casein, hydrolyzed casein (CPH), whey, hydrolyzed whey (WPH), and hydrolyzed blend (HB). Asterisks indicate the significance of each treatment relative to controls (* *p* < 0.05; ** *p* < 0.01; *** *p* < 0.001). Data show mean ± SEM.

GE for the DMSO–hydrolyzed soy diet was not altered by the pharmacological modulators (Figure 5A). Transit was slowed by loperamide for the DMSO–hydrolyzed soy control diet treatments at 9 h and 12 h (Figure 5B). Slowing of GI transit for casein was reversed by prucalopride (9 h), but slowing for whey was not affected by prucalopride. Loperamide affected transit for neither casein nor whey treatment conditions. Rapid GE for the HB (9 h and 12 h) was slowed 80–90% by loperamide (Figure 5A). Furthermore, the relatively shorter transit (9 h) for the HB compared with the intact proteins was also inhibited by loperamide (Figure 5B). No significant drug effects were detected for cecum to colon transit. The modulatory drugs did not alter bead transit through the colon on any of the diets.

Overall changes in movement of GI contents induced by the dietary and pharmacological treatments are summarized in Table 2.

## 4. Discussion

The initial finding of this study was that casein (intact) slowed GI transit compared with hydrolyzed soy, as expected, and set a comparative benchmark with the other milk proteins. The slowed GI transit by casein can be largely attributed to delayed GE. The slowed GI transit for whey, however, was not due to delayed GE but rather is likely to be localised to slower bead movement through the SI. Contrary to our hypothesis, CPH and WPH proteins did not reduce GI transit time relative to their intact counterparts (or hydrolyzed soy) and once in the colon, the transit was not different from the other proteins studied.

### 4.1. Hydrolyzed Soy Protein

GE and GI transit and fecal output measurements for the hydrolyzed soy treatment were similar to those previously reported using this method in aged rats [36]. Because loperamide was effective at inhibiting GI transit, this suggested that mu opioid receptors were available, arguing against any dietary soymorphins specific to these receptors having a strong inhibitory influence on motility. The corresponding decrease in food intake detected for soy by loperamide was also reported previously [36].

### 4.2. Casein Protein

Delayed GE of solids and SI transit for casein compared with hydrolyzed soy is consistent with a previous rat study on the effect of casein relative to soy protein isolate on transit of the liquid phase of chime, in which GE was slowed at 1 h and SI transit slower after a further h [41]. Our finding that GE is slower for casein than for whey or WPH is consistent with decreased food intake on the casein diet (at 14 days) compared with the other diets.

Reversal of slowed GI transit for casein (compared with soy) by prucalopride suggests that peptides with pharmacological actions are present in the casein and that these likely inhibited neural control of GI motility. Because transit by casein was not further inhibited by loperamide, this implicates endogenous opioid agonist peptides such as the β-casomorphins in the slower SI and LI transit with casein (compared with soy). Such peptides would have been released from the casein during gastric digestion and might therefore also have contributed to the delayed GE for casein. The decrease in food intake could be due to delayed GE or opioid antagonist peptide release during digestion [42,43].

### 4.3. Whey Protein

The GE of solids for whey was markedly faster than for casein, but SI transit was slower for whey than that for either hydrolyzed soy or the HB. The relatively slower GI transit for whey was therefore not due to delayed GE as for casein, but rather was localised to the SI. This provides new evidence that whey did in fact slow SI transit compared with casein and demonstrates this as independent from GE.

The lower GI effect of loperamide in decreasing fecal output indicates that an opioid agonist was able to slow fecal movement for whey in the distal colon–rectum region. It is possible that the altered fecal output by loperamide for whey in vivo involved additional effects on fluid and electrolyte secretion [44].

### 4.4. Hydrolyzed Casein

Similarly to casein, GE was also slowed for CPH. This may be attributed to it only being 7.6% hydrolyzed such that significant peptide release could still occur during digestion. GI transit was not altered by CPH, compared with soy or casein, but unlike casein, loperamide was effective at inhibiting GI transit for animals fed the CPH diet. This suggests that the CPH lacked sufficient opioid agonist activity to significantly slow transit. This was likely due to the partial hydrolysis used to generate the CPH, resulting in partial release of some small peptides (e.g., opioid agonist) that are then lost during processing. The remaining peptides released during digestion would then be insufficient to elicit a significant transit slowing effect. Although hydrolysis of the casein protein did not increase GE or GI transit compared with the casein overall, prucalopride increased fecal output, suggesting that it had been slowed on this diet in the distal colon–rectum region. The ability of the serotonin agonist to further increase fecal output suggests that it reversed a partial inhibitory effect of the CPH on colonic motility. Our results differ from those previously reported for CPH (DH 27%), in which hydrolysis increases transit compared with casein in rats [8], although trended similarly over 12 h. The degree of casein hydrolysis was positively associated with slowed GI transit. CPH is considered a rich source of opioid receptor ligands, in particular the beta casomorphins which activate the mu receptor [45], slowing GI transit in the rat compared with whey [7]. However, an effect of hydrolysis on GE and transit in our study was not detected under normal conditions. However, when challenged using pharmacological modulators, differences in responsiveness to these became evident, suggesting different bioactive peptides between the two casein forms. This suggests that during digestion, peptides with mu opioid receptor activity were released from casein, but fewer from CPH.

### 4.5. Hydrolyzed Whey

GE and GI transit effects of WPH were similar to those for hydrolyzed soy, rather than being slowed as for casein. No delay in GE occurred as a result of whey hydrolysis. This is consistent with the knowledge that casein aggregates in the stomach where it is digested by physiological enzymes, whereas being soluble proteins, whey and soy pass rapidly through the stomach and undergo digestion by pancreatic enzymes [3].

Despite the apparent similarity between GI transit effects of the WPH diet compared with soy, fecal output was increased compared with soy (and native whey). There was no corresponding increase in food intake for whey, possibly due to the short term satiating effect attributed to whey proteins in some studies [46]. Taken together, the rapid GE and increased fecal output in response to WPH suggest that this would be a useful ingredient in a functional food or beverage for the elderly to enhance GE and reduce constipation.

Loperamide was effective at inhibiting overall GI transit only if the whey was hydrolyzed. This suggests that the WPH lacked mu opioid agonist activity to inhibit transit. Loperamide did however decrease fecal output to a similar extent for both intact and WPH forms (both by 35%). The effect on fecal output being common to both forms of whey suggests an alternative mechanism was involved, probably localised to the rectum.

### 4.6. Hydrolyzed Whey–Casein Blend

The HB produced the most rapid GI transit of the milk proteins studied with rapid GE a contributing factor. This is consistent with these proteins having undergone a greater degree of hydrolysis compared with CPH and WPH. The HB was similar to soy in transit attributes imparted, in particular that both had increased SI transit compared with casein (and whey). The rapid SI transit rate and increased fecal output (compared with CPH) attributes of the WPH component appear to dominate. We recognise that this might also be due to relative higher level of hydrolysis for the HB. Relative to casein, the HB produced the greatest increase in transit through the SI of the proteins studied, an effect partly attributed to more complete GE. Our results suggest that the HB might also be a useful ingredient in a functional food or beverage for the elderly to enhance GE, which can slow with healthy aging [11].

Our findings agree with a study in which a partially hydrolyzed 60:40 whey–casein-based formulae accelerated transit of milk and stools compared with its non-hydrolyzed cow milk protein counterpart [9].

### 4.7. Modulators and Mechanisms

All three hydrolyzed ingredients studied were sensitive to opioid agonist modulation in slowing transit. This suggests that they either do not contain significant milk-derived opioid peptide concentration or contain alternate components that speed transit. The heightened sensitivity of HB-fed rats to a mu opioid agonist suggests that milk-derived opioid peptides that inhibit GI transit via the mu opioid receptor are not present in significant amounts. The greater degree of hydrolysis for the whey and casein in the blend compared with the CPH or WPH means that different peptides are likely present that confer pro-motility actions. These findings are consistent with the idea that industrial hydrolysis of milk proteins precludes subsequent release of peptides that occurs during the normal digestive process in the GI tract [17].

The shortened GI transit for the HB suggests absence of motility slowing peptides possibly together with additive pro-motility actions of peptides in the mixture. This is supported by the greater fecal output for the HB compared with soy when both received prucalopride treatment. In contrast, casein was sensitive to a serotonin agonist (5-HT_4_) in reversing the slowed transit.

## 5. Conclusions

In summary, the expected slower GI transit for casein relative to hydrolyzed soy provided a comparative benchmark. Lack of loperamide modulation of the slowed casein effect corroborated opioid agonist activity for casein peptides in the SI. Reversal of the casein-induced slow transit by prucalopride showed that a 5-HT_4_ agonist could counter this effect. Our findings provide new evidence that whey protein slows SI transit compared with hydrolyzed soy and indicate that this is independent of GE. This suggests that whey protein may slow small intestine transit to allow time for increased nutrient absorption to occur without slowing GE, which would be helpful for improved nutrition in the elderly. Further studies would be required to verify whether nutrient uptake has been affected. Despite the inability of the CPH or WPH to shorten transit compared with their intact counterparts, fecal output was increased upon whey hydrolysis. Furthermore, opioid agonist inhibition of transit on CPH or WPH diets suggests little opioid agonist activity by peptides in these hydrolysates. The increased the rate of GI transit from stomach to colon relative to casein by WPH and the HB suggests that their incorporation in dairy formulations may be beneficial in treating functional GI disorders involving constipation, particularly for the aged.

## Figures and Tables

**Table 1:** Animal food intake and fecal output.

Protein	Food Intake (g)	*n*	Fecal Output (g)	*n*
**Control**				
Hydrolyzed soy	23.5 ± 2.0 ^a^	16	3.3 ± 0.6 ^a^	15
Casein	21.8 ± 1.8	10	3.9 ± 0.4	10
Whey	22.2 ± 1.8	10	3.5 ± 0.4 ^b (^*^a)^	10
CPH	22.4 ± 1.8 ^b^	9	2.8 ± 0.4 ^c^	8
WPH	24.5 ± 1.8 ^c^	9	4.9 ± 0.4 ^d (^**^a) (^*^b) (^**^c)^	9
HB	25.5 ± 1.8 ^d^	9	4.3 ± 0.4 ^e (^*^c)^	9
**Loperamide**				
Hydrolyzed soy	20.0 ± 1.8 ^(^*^a)^	15	2.9 ± 0.4	15
Casein	20.4 ± 1.8	9	3.1 ± 0.4	9
Whey	19.5 ± 1.8 ^(^*^a)^	12	2.2 ± 0.4 ^(^*^a) (^*^b)^	12
CPH	18.2 ± 1.8 ^(^**^a) (^*^b)^	9	2.7 ± 0.4	9
WPH	18.4 ± 1.8 ^(^**^a) (^***^c)^	11	3.2 ± 0.5 ^(^**^d)^	11
HB	20.4 ± 1.8 ^(^**^d)^	9	3.0 ± 0.5 ^(^*^e)^	9
**Prucalopride**				
Hydrolyzed soy	24.1 ± 1.8	15	3.6 ± 0.4 ^f^	15
Casein	21.3 ± 1.5	7	3.5 ± 0.4	7
Whey	23.6 ± 1.8	8	3.7 ± 0.4	8
CPH	23.6 ± 1.8	8	4.1 ± 0.4 ^(^*^c)^	8
WPH	22.8 ± 1.8	7	4.1 ± 0.3	5
HB	23.2 ± 1.8	8	4.8 ± 0.4 ^(^*^f)^	8

Data shown after 21 days on protein diet with drug treatment over last 7 days. CPH, casein protein hydrolysate; WPH, whey protein hydrolysate; HB, 60:40 whey/casein hydrolyzed blend. Data show mean ± SEM. Asterisks in brackets indicate statistical significance (* *p* < 0.05, ** *p* < 0.01, *** *p* < 0.001) of the differences among diet and drug treatments compared with those indicated by the superscript letters.

**Table 2:** Summary of altered GI function.

Protein	GE	SI Transit	LI Transit	Fecal Output
Hydrolyzed soy	fast	fast (↓LP)	fast (↓LP)	ND
Casein	slow	slow (↑PC)	slow	ND
Whey	>casein	<soy	mid	(↓LP)
CPH	slow	mid (↓LP)	mid	(↑PC)
WPH	fast	fast (↓LP)	fast (↓LP)	>whey & soy (↓LP)
HB	fast	fast (↓LP)	fast (↓LP)	>CPH

GI, gastrointestinal; GE, gastric emptying; SI, small intestine; LI, large intestine; CPH, casein protein hydrolysate; WPH, whey protein hydrolysate; HB, 60:40 whey–casein hydrolyzed blend; LP, loperamide; PC, prucalopride; ↓, slowed by; ↑, accelerated by; ND, not determined.
